# Bee++: An Object-Oriented, Agent-Based Simulator for Honey Bee Colonies

**DOI:** 10.3390/insects8010031

**Published:** 2017-03-10

**Authors:** Matthew Betti, Josh LeClair, Lindi M. Wahl, Mair Zamir

**Affiliations:** 1 Department of Applied Mathematics, Western University, London, ON N6A 5B7, Canada; matthew.betti@gmail.com (M.B.); jlecla3@uwo.ca (J.L.); lwahl@uwo.ca (L.M.W.); 2 Department of Medical Biophysics, Western University, London, ON N6A 5B7, Canada

**Keywords:** agent-based simulation, honey bees, Apis melliflora, population dynamics, colony collapse disorder

## Abstract

We present a model and associated simulation package (www.beeplusplus.ca) to capture the natural dynamics of a honey bee colony in a spatially-explicit landscape, with temporally-variable, weather-dependent parameters. The simulation tracks bees of different ages and castes, food stores within the colony, pollen and nectar sources and the spatial position of individual foragers outside the hive. We track explicitly the intake of pesticides in individual bees and their ability to metabolize these toxins, such that the impact of sub-lethal doses of pesticides can be explored. Moreover, pathogen populations (in particular, *Nosema apis*, *Nosema cerenae* and *Varroa* mites) have been included in the model and may be introduced at any time or location. The ability to study interactions among pesticides, climate, biodiversity and pathogens in this predictive framework should prove useful to a wide range of researchers studying honey bee populations. To this end, the simulation package is written in open source, object-oriented code (C++) and can be easily modified by the user. Here, we demonstrate the use of the model by exploring the effects of sub-lethal pesticide exposure on the flight behaviour of foragers.

## 1. Introduction

Due to the importance of honey bees, both ecologically and agriculturally [1,2,3,4], and because of their decreasing populations on a global scale, honey bees have been the focus of a large body of research [5,6,7,8,9,10,11,12,13]. The recent problem of colony collapse has led to a surge in research efforts into honey bee colony dynamics and the stresses they face. Efforts to understand the causes underlying honey bee decline are impeded by complex colony dynamics, along with the numerous interactions bees have with their local ecosystem; these factors create a system with multiple entwined variables, often confounding causality [1]. In order to gain insight into the problem of colony collapse and into the impact of honey bees as key players in their local ecosystem more generally, the suspected causes of colony collapse are often studied in isolation. In the last fifteen years, both experimental and theoretical studies have looked into the effects of factors such as competing hives [14], parasites [12,15,16,17], food stores [18], and pesticides [8]. More recently, the combined effects of parasites and pesticides have been addressed experimentally [19].

Due to the complexity of the problem, mathematical modelling has emerged as a valuable tool for exploring the dynamics of honey bee colonies; in particular, modelling approaches are well-suited for understanding the combined effects of multiple stressors on the colony. Several mathematical studies have addressed the compounding effects of pesticides and parasites on a honey bee colony [15,20,21,22,23]. In [15,20], the effects of acute bee paralysis (vectored by the *Varroa* destructor) are studied in combination with pesticides and seasonality respectively. Pettis et al. predicted that an exposure to pesticides will increase the susceptibility of *Nosema ceranae* [23], which was shown experimentally by [22]. The effects of pesticide use in the local environment in the context of honey bee protection policy is studied, through models, in [21]. Others have further broadened the scope to account for the combined effects of pesticides and parasites, as well as a lack of biodiversity in the local environment [24]. Our previous studies have focused on the combined effects of temperature, seasonality and disease [13,25].

These combined effects have also been synthesized into software simulation packages, studying in silico honey bee colonies in a wide range of environmental scenarios [26,27]; this individual-based approach has proven useful for many ecological models [28]. The honey bee software packages are agent-based and include temperature data, stochastic behaviour, and seasonality. Also included in [26] is *Varroa* transmission, as well as possible human intervention in the context of care, or honey harvesting. Pesticide exposure is modelled as an increase in the death rate of foraging bees. In [27], the basic dynamics of a honey bee colony are modelled in depth.

We have developed a software package, Bee++, that can accurately model a honey bee colony in a spatially-explicit landscape, in which individual foragers can be tracked for the entirety of their trips away from the hive. Building on previous approaches, our model includes age, caste, food stores, temperature data and pathogens. In addition, our proposed model relaxes the typical simplification that pesticides can only affect the death rate of bees and allows for explicit tracking of toxin levels within individual bees. As a consequence, our model also allows individual bees to detoxify over time by modelling known mechanisms [29,30]. This allows Bee++ to be used to simulate and study the long-term effects of pesticide exposure at non-lethal doses, as well as to simulate the compounding effects of different pesticides or prolonged exposure on honey bee physiology. The software package is highly configurable and developed on an open source platform. The individual parameters within the model may be varied by the user, and moreover, the functional form of the processes involved (e.g., recruitment to foraging, detoxification rate) can be modified. In what follows, we describe the algorithms underlying Bee++, with particular attention to functions and parameters that are configurable by the user. Our aim is to provide a platform that can be modified and extended to address a number of research questions. In what follows, we examine the effects of long-term pesticide exposure on the navigation abilities of foragers, primarily to illustrate the use of Bee++.

## 2. Model

Bee++ (http://www.beeplusplus.ca) consists of a stochastic, agent-based model for a honey bee colony and the surrounding environment. The model includes three classes of bees: hive bees, whose sub-classes consist of juveniles, nurses and maintenance workers; foragers, whose sub-classes consist of scouts, nectar carriers and pollen carriers; and drones. The egg-laying function of the queen is modelled but, since Bee++ currently simulates the dynamics of a single colony, subsequent new queens produced in the hive are omitted.

Bee++ keeps track of the age of each bee, its current role, as well as any diseases it carries and its level of toxin exposure at any time. The simulation also monitors the state of the brood, the food stores in the hive (both pollen and honey, separately) and their levels of contamination by pesticides, fungicides or pathogens.

The environment external to the hive is modelled by the user as a spatial grid, in which some elements may contain nectar or pollen sources, and/or sources of pesticides or pathogens. These sources may be constant or may vary with the season as defined by the user. Within this grid, the position of each bee at each time step is tracked and updated as described in the sections to follow.

**Figure 1:**
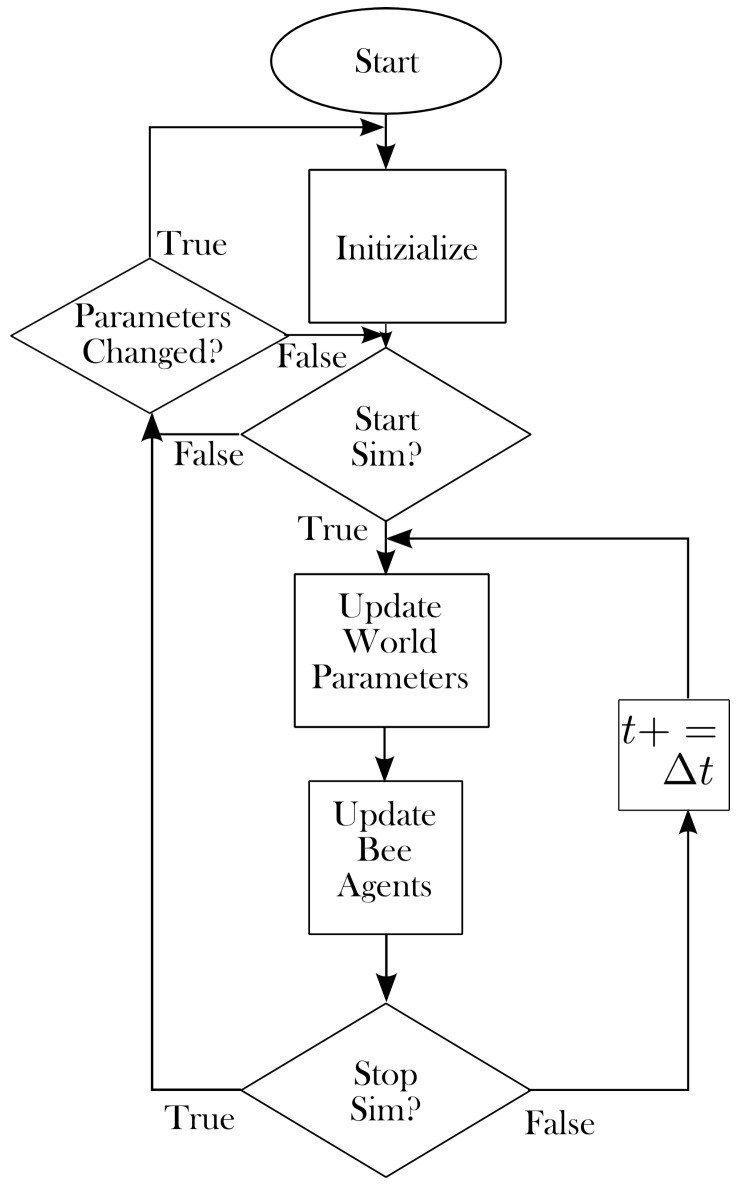
Flow diagram of the main program loop.

**Figure 2:**
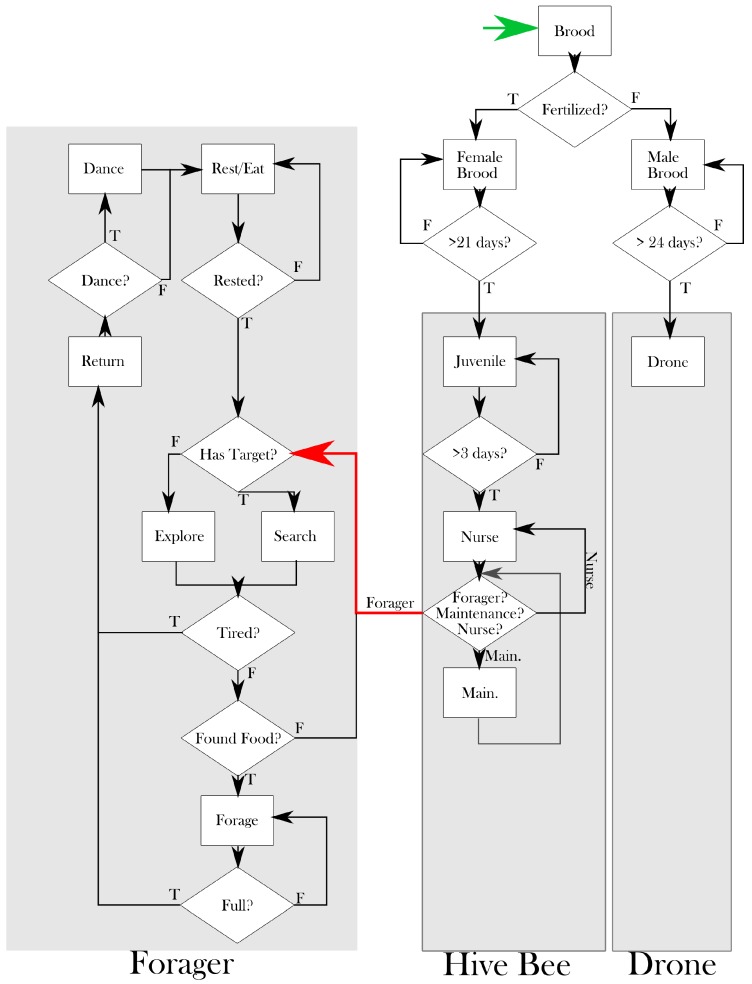
Flow diagram of the three main bee classes and their possible decisions in one time step. This diagram is not exhaustive, but intended to be illustrative of the main decision tree for each of the three main classes: **drones**, **hive bees**, and **foragers**. These classes of bees are further divided into different states, which changes the behaviours and parameters associated with each bee. The **green arrow** begins the cycle for one bee agent. The queen lays an egg, which then gets added to the **Brood**. The Brood are either fertilized (female) or not (male). After 21 days, the fertilized brood will emerge and become **juveniles**. **Hive bee:** The **juveniles** are responsible for cleaning cells. After three days, the hive bee juvenile will become a **nurse**. The nurses are responsible for caring for the brood, ensuring their survival. At each time point, the nurse will decide to become a **maintenance** bee, remain a nurse or become a **forager**. The **maintenance** bees are responsible for hive repair, security and other duties around the hive that are specifically *not* caring for brood. Maintenance bees can also revert to nurse bees if there is a need, or be recruited (**red arrow**) to the forager class. The probability of the bee choosing one of these three states is dependent on the needs of the hive, the bee’s age and many other factors (pesticide exposure, disease, weather etc.). The **forager** class is typically an ‘end of life’ class. Bees recruited to forager will leave the hive. If they have a target, the bee will **search** out the target. If the bee does not have a target or loses its target, it will **explore**. A bee who is **tired** (i.e., runs out of energy) will **return** to the hive. If a bee has **found food**, it will begin to **forage** in that patch. When the bee is **full**, it will **return** to the hive. In the hive, the bee may decide to **dance** to relay the location of a food source to other bees. Whether or not a bee dances, she will always **rest**. Once rested, the forager will again leave the hive to forage. **Drones:** After 24 days, an unfertilized egg will develop into a drone. Currently, drones in Bee++ stay in the hive and consume resources until they die or are ejected by the females. At each state, the bee has a probability of death, which has been omitted from the diagram for clarity.

As a brief overview of the algorithm, at each time step the time-dependent parameters (e.g., environmental temperature) are updated, and then the decisions of each bee are considered. The number of brood is only updated once per day for simplicity and computational consideration. The number of brood that survive is dependent on the current food stores and amount of care available. The decisions of the hive bees depend on a number of parameters, namely temperature, infection, food stores, age and toxicity. The hive bees are able to transition between juveniles, nursing duties, maintenance duties and foraging based on the aforementioned parameters. Once recruited to foraging, the bees begin exploring the environment looking for food. In this way, they are able to transmit infection and pesticides from the environment back to the hive. They are also able to recruit new bees to particular patches of food that they have found. The navigational abilities of the foragers, along with their ability to survive, recruit and carry food are dependent mainly on the parameters mentioned above. Flow diagrams for the main program loop and for the three main classes of bees are provided in Figure 1 and Figure 2, respectively. Details for the various processes follow.

### 2.1. Bees

Hive bees: Hive bees are modelled as agents, which, at each time step, make probabilistic decisions based on their age, the current demography of the colony and the state of the surrounding environment. The most important of these decisions is whether or not to be recruited to foraging duties. The recruitment rate increases with the age of the bee and is reduced if the temperature is not appropriate for foraging or if the fraction of bees already foraging is optimal. Specifically, a hive bee of age *a* begins watching a waggle dance (with the possibility of being recruited to also forage from that patch) at rate

(1) $$\begin{array}{c} p_{W}=\alpha \left(\frac{4\left(T-T_{1}\right)\left(T_{2}-T\right)}{{\left(T_{1}+T_{2}\right)}^{2}}\right)\left(\frac{a}{k+a}\right)\left(1-\sigma \frac{F_{T}}{N}\right) \end{array}$$

where *α* is the base rate of recruitment, *k* denotes the age at which a hive bee has a 50% chance of being recruited, $F_{T}$ is the total number of bees currently foraging and *N* is the total working population in the colony. The factor 1/σ thus represents the fraction of foragers required to halt recruitment through social inhibition [31,32,33]. $T_{1}$ and $T_{2}$ are threshold temperatures such that foraging stops if the environmental temperature $T<T_{1}$ or $T>T_{2}$, and foraging peaks at $T=(T_{1}+T_{2})/2$. This is consistent with experimental findings that there is a peak foraging temperature [34] and that foraging decreases away from this optimal temperature [35,36]. Note that Equation (1) represents the default recruitment function in Bee++; not only can each parameter be set by the user, but the form of the function itself can be reconfigured as needed through straight-forward modifications to the open source code.

At some point a hive bee foregoes other duties and waits to watch a forager as it returns to the hive and begins a dance. The hive bee then inherits the target coordinates and carrier type (pollen or nectar) of the forager. If there is more than one forager dancing during the time that the hive bee is watching, the hive bee has equal chance of following any of the current dances, or becoming a scout.

Foragers: Foragers are divided into subclasses: scouts, recruits (working foragers), resting foragers and dancers [37]. Each forager follows the directions of a waggle dance to the target food patch, but does not follow these directions perfectly; thus, movement is dictated by a biased random walk toward the destination.

We acknowledge that many strategies may be used by honey bees when foraging, and a number of movement models have been proposed in the literature [37,38,39]. Due to the resolution of the spatial grid in our model, however, we found a biased random walk sufficient. A finer grid, in which individual plants could be resolved, would be better modelled by a Lévy flight [40].

In the biased random walk, the inherent error in following directions is given by parameter *ε*. The probability of travelling in the correct vertical direction is given by

(2) $$\begin{array}{c} p_{V}=\left(\frac{d_{V}}{d_{V}+d_{H}}\right)(1-\varepsilon )+\frac{\varepsilon }{4} \end{array}$$

where $d_{V}$ is the vertical distance between the forager and its target, and $d_{H}$ is the horizontal distance between the forager and its target. In other words, if the forager is at coordinates $\left(x_{F},y_{F}\right)$ and the target food patch is at coordinates $\left(x_{T},y_{T}\right)$, then

(3) $$\begin{array}{cc} d_{V} & =|y_{T}-y_{F}| \end{array}$$

(4) $$\begin{array}{cc} d_{H} & =|x_{T}-x_{F}|. \end{array}$$

Similarly, the forager travels in the correct horizontal direction with probability

(5) $$\begin{array}{c} p_{H}=\left(\frac{d_{H}}{d_{V}+d_{H}}\right)(1-\varepsilon )+\frac{\varepsilon }{4}. \end{array}$$

This leaves a probability $\frac{\varepsilon }{4}$ of travelling in each of the two wrong cardinal directions. Note that the error parameter, *ε*, is set by the user and can be a function of the toxicity level in an individual bee, such that pesticides that may interfere with navigation can be simulated. The parameter *ε* may be affected by pesticides ingested by the forager, and this is modelled explicitly as

(6) $$\begin{array}{c} \varepsilon =\varepsilon_{base}+\varphi X \end{array}$$

where *X* is the toxicity level of the bee, *φ* is a configurable parameter and $\varepsilon_{base}$ is the natural error in a honey bee’s navigation.

In addition, this randomness in movement is necessary to allow foragers to find new sources of food. In fact, our simulation is set up in such a way that if a forager comes across a viable food source before reaching its target, it will update its target to the patch it has just found.

Foragers will eventually give up on their target if they are looking for too long without successfully finding the target food source [41]; this search time limit in our model is given by $t_{S}$. Furthermore, bees who have been out of the hive for time $t_{H}$ will attempt to return home before death from exhaustion. Foragers are considered lost if they die a distance $d_{L}$ from the hive or from any food patches.

The duty of a scout is solely to find new sources of pollen or nectar [42]. Therefore, they do not have a target, and thus, by setting $d_{V}=d_{H}=0$, we are able to allow scouts to diffuse through the environment until they reach a viable food source. Put simply, a scout’s movement is governed by a random walk. Once a food source is found, the scout will use a biased random walk to navigate back to the hive and back to the food source in the future.

We assume that a forager always carries a full load of either nectar or pollen back to the hive and that the time it takes to extract the nectar/pollen from the source depends solely on the properties of the source. When they get back to the hive, food stores are updated for both nectar, pollen and any contaminants carried, as described further in Section 2.2. Upon their return, foragers also decide to dance with (currently) fixed probability, $p_{Dance}$.

Drones: Our model also accounts for drones in the hive. The details of drone dynamics can be found in Appendix A.1.

The brood: The queen has substantial, if not full, control over the number of fertilized eggs she lays [43,44]. In general, the number of eggs laid is seasonally dependent [45]. In addition, the queen’s health depends on food availability, and thus we assume the number of eggs she may lay per day depends on the food available to her. Therefore, we model the number of eggs laid per day as

(7) $$\begin{array}{c} L=L_{B}\;\min\left(\frac{f_{P}}{f_{P}+b}e^{-E_{1}{\left(t-\hat{t}\right)}^{2}},1\right)\;, \end{array}$$

where $\hat{t}$ is the day of the year on which the queen lays the most eggs, and $E_{1}$ is a constant determining the egg laying season of the queen. The variable $f_{P}$ is the amount of pollen available in the hive, while *b* is a shape parameter (amount of pollen at which the number of eggs laid is half maximum). $L_{B}$ is a randomly generated number to simulate the decision-making of the queen. Specifically, $L_{B}$ is Poisson-distributed with a mean value set by the user, which is the mean number of eggs laid per day at the peak of the season, if pollen stores are plentiful. After *L* is computed, the number of fertilized eggs is determined by a binomial random variable; the probability that an egg is fertilized is set by the user.

For computational considerations, the brood is not considered on an individual level. Instead, the number of brood cells that survive in one day is dependent on the amount of pollen available for consumption and the amount of care that can be provided by the nursing bees [10]. This fraction is denoted the survivability function, *S*, and is defined as

(8) $$\begin{array}{c} S=\left(\frac{f_{P}}{f_{P}+b}\right)\left(\frac{N_{N}}{N_{N}+w}\right) \end{array}$$

where $N_{N}$ is the number of bees currently nursing and *w* is a shape parameter (number of nurses for which half the brood survives). As before, we note that the functional forms of both *L* and *S* are configurable; we describe here the default functions currently implemented.

The worker brood are capped at eight days by default, a parameter value motivated by [46]. Between this day and when the pupae emerge, they survive off the food provided during their larval stage and are considered well protected. Surviving worker bee pupae emerge 21 days after egg laying [47], and surviving drone pupae after 24 days of development. Three days after emerging, the workers begin their nursing duties [48]. We emphasize that we choose these values as defaults as they correspond with parameter values in the literature. All parameters can be modified to simulate different scenarios.

Toxicity: Each bee is assumed to have a level of toxicity. Toxicity increases when toxins (such as pesticides) are ingested, and decreases naturally through detoxification mechanisms. These mechanisms are poorly understood [49], but it is known that certain fungicides hinder certain mechanisms (namely P450 detoxification) [30]. In order to simulate these particular interactions, Bee++ is configured to account for one type of pesticide, *P*, and one type of fungicide, F, which interact in their effects on bee toxicity, such that:

(9) $$\begin{array}{c} \frac{dX}{dt}=\gamma_{N}C_{P}-x_{D}\frac{X}{\gamma_{N}C_{F}+X+1}. \end{array}$$

Here, *X* is the toxicity level of the bee; $\gamma_{N}$ is the amount of food ingested in one time step; $C_{P}$ is the concentration of pesticide in the food stores; $x_{D}$ is the base rate of detoxification in a honey bee; and $C_{F}$ is the concentration of fungicide in the food stores. Thus, the detoxification rate is reduced by high levels of fungicide in the food stores. Of course, as more information becomes available this can be modified to incorporate many types of interactions between a multitude of different toxins. The concentrations $C_{P}$ and $C_{F}$ depend on the influx of contaminated food from different sources. Each source, *i*, may have a different concentration of toxin present, denoted by ${\hat{C}}_{P,i}$ and ${\hat{C}}_{F,i}$; these are again configurable. The parameter $x_{D}$ is generally difficult to measure, but recent studies have been able to measure the half life of pesticides within a honey bee [50,51], $t_{1/2}$, and from this value, we can calculate a metabolization rate, $x_{D}$, through the conversion $x_{D}=\ln\left(2\right)/t_{1/2}.$

Unlike previous agent-based models, or even general mathematical models for honey bee colony dynamics, the above method of tracking pesticides allows for exploration of the effects of toxins on the colony in both a spatially explicit and toxicologically explicit way. Previous models have simulated the effects of pesticides only by increasing the natural death rate of the foragers [10,11,13,15,18,20,21,52]. Bee++ allows us, in addition, to track the effects of pesticides on, for example, a bee’s ability to navigate its environment or potentially the effects on recruitment by modifying Equations (1), (2), or (5), or parameter *α*.

Death rates: The death of bees is also handled stochastically. The probability of death at any given time is dependent on many factors, and in this model we simulate death as a function of temperature, disease, toxicity, food availability and natural causes.

Bees outside the hive are subject to death rate $d_{O}=d_{n}+d_{d}+d_{T}+d_{Tox}$, where each term is defined below. The natural death rate, $d_{n}$, is given by

(10) $$\begin{array}{c} d_{n}=C\left(1+\frac{K}{K+f_{N}}\right)\frac{{\left(a-a_{opt}\right)}^{2}}{a_{opt}^{2}} \end{array}$$

where $a_{opt}$ is the optimal age of a forager (when it is the strongest), $f_{N}$ is the amount of nectar available in the colony, and *C* and *K* are scaling parameters. Thus, the natural death rate increases quadratically for young or old bees and can be increased by a factor of two if insufficient nectar is available.

The death rate is also sensitive to the environmental temperature, *T*, such that:

(11) $$\begin{array}{c} d_{T}=\left\{\begin{array}{cc} (1-Ae^{-J{\left(T-T_{I}\right)}^{2}})\; & d_{T}>0\\ 0\; & \mathrm{otherwise}. \end{array}\right. \end{array}$$

Here, $T_{I}$ is the ideal environmental temperature for honey bees; *A* and *J* are scaling parameters.

The death rate due to toxicity depends on the toxin (or combination of toxins) within the bee and is proportional to a user-defined combination of toxins

(12) $$\begin{array}{c} d_{Tox}=K_{T}X \end{array}$$

where $K_{T}$ is a scaling parameter that would depend on the specific toxin being studied and its associated LD${}_{50}$ value and *X* is the toxicity of the individual.

The disease-related death rate is disease dependent and configurable. If a bee suffers from multiple inflictions, we assume the effects are additive. For example, nosemosis induced by *N. cerenae* has been shown to double the death rate of honey bees [53], and thus, for this particular disease, we have

(13) $$\begin{array}{c} d_{d}=d_{n}. \end{array}$$

Bees inside the hive suffer from the same natural death rate (Equation (10)), with a different scaling parameter *C* due to the safety the hive provides. They are also subject to death due to differences in temperature, although temperature affects these bees in a different way.

Specifically, bees work to keep the temperature inside the hive constant at 35 ${}^{\circ }$C by either fanning their wings to cool, or expending metabolic heat to warm the hive [54]. With this information, we posit that the effects of temperature within the hive are tied to the number of bees within the hive [55]. The number of bees needed to maintain a constant temperature within the hive is thus modelled to be proportional to the difference between the ambient temperature and the target temperature. In other words, the farther away the ambient temperature is from the ideal, the more bees are required to produce the necessary metabolic heat to maintain a constant temperature. Therefore, inside the hive, we define temperature-related death as

(14) $$\begin{array}{c} d_{TH}=\frac{C_{1}\left|T-T_{I}\right|}{C_{2}+C_{3}N_{H}} \end{array}$$

where $C_{1}$, $C_{2}$ and $C_{3}$ are constants that can be adjusted and $N_{H}$ is the total number of bees within the hive.

When the temperature outside the hive drops below the threshold, $T_{W}$, all bees attempt to return to the hive and begin over-wintering behaviour (i.e., all bees act to maintain brood and control the temperature of the hive). Drones are cast out of the hive, as during winter they are a detriment to survival [56].

### 2.2. Food Stores

Bee++ keeps track of the amount of pollen, $f_{P}$, and honey (derived from nectar), $f_{N}$, at time *t* in the hive. The average concentrations of pesticides and fungicides, $C_{P}$ and $C_{F}$, in these food stores are also tracked.

For simplicity, food stores are assumed to be consumed at a constant rate by bees that are present in the hive at any given time. This means that individual bees do not make a decision to eat, but are assumed to find time within each time step to eat, based on the consumption rate for their caste $\gamma_{j}$. Therefore, the amount of pollen is reduced by $\Delta t\gamma_{B}B_{U}$, while the amount of honey is reduced by $\Delta t(\gamma_{H}H+\gamma_{F}F_{R})$ in each time step Δt, where $B_{U}$ is the total population of uncapped brood, $F_{R}$ is the number of resting foragers and *H* is the total hive bee population. The amount of pesticide removed from the food stores per bee, as the bees consume pesticides with the food, is given by $C_{P}\gamma_{j}\Delta t$, where *j* represents the particular caste of the bee eating; fungicides are treated analogously. In its present form, Bee++ assumes that pesticides do not decay within the food stores, or in the environment.

When a forager returns to the hive from the environment, it will add its load, $c_{P}$ or $c_{N}$, to the pollen or honey stores, respectively. The amount of pesticide added to the food stores from source *i* is $c_{P}{\hat{C}}_{P,i}$; again, fungicides are treated analogously.

The average water content of honey is approximately 20% [57], whereas the water content of nectar can vary anywhere between 20% and 95%, depending on the plant of origin [57]. This gives a median value of 57.5% water content for nectar, which is in close agreement with the water content given for alfalfa and clover (55% water concentration) given by Wilson et al. [58]. With these numbers, we can deduce that the sugar concentration of nectar is approximately 45%, and the sugar concentration of honey is approximately 80%. Using these numbers, we can convert the weight of nectar in grams to the weight of honey in grams via

(15) $$\begin{array}{c} W_{h}=0.54W_{n} \end{array}$$

where $W_{h}$ is the amount of honey by weight and $W_{n}$ is the amount of nectar by weight. This is necessary, as foragers collect nectar, which is then converted to honey in the hive.

### 2.3. Environment

The environment is represented by an N×N grid of space with which the bees can interact. Each element of the grid is either empty (meaning a bee may fly through it without any interactions), a pollen source, a nectar source, or a mating patch as defined by the user. One cell (typically the centre) is designated as the hive. The pollen and nectar sources have the ability to be time-dependent to model seasonality, and may be depleted as the resource is consumed by the bees. The cells may also harbour pesticides or fungicides, which are transferred to foragers who interact with the patch, who in turn contaminate the hive food stores when returning to the hive with pollen or nectar. Again, for computational tractability, we assume that the pesticides are well mixed in both the sources of food, and in the food stores in the hive.

In terms of the effects of pollination on the flowers, we track how many bees return to the same plant type for two (or more) consecutive trips. With each consecutive trip, we assume the bee is able to pollinate more flowers and thus increase the potential fruit yield for the patch. In this way, Bee++ is able to simulate not only honey bee dynamics, but also the effects of honey bee colonies on a surrounding environment.

The environmental patches are also able to harbour pathogens and pesticides, which can then be transmitted to forager bees and brought back to the hive. Moreover, in the case of certain pathogens, infected foragers may leave parasites on a plant, resulting in infection of subsequent visitors. The amount of pollen and/or nectar available at a given patch is seasonally dynamic, allowing for studies of biodiversity as a potential stressor.

The default time step of Bee++ is on the order of minutes. This gives Bee++ the ability to track the foraging trips of individuals, which, on average, last 6–7 min for healthy bees [42,59]. The default spatial scale is on the order of square meters per spatial element of the grid. Because of the fixed flight speed of the bees, the spatial scale and time step are linked. Large areas can be modelled by scaling back the resolution of the map (i.e., allowing for larger cells), and in this case the time step should be adjusted to days or weeks, such that bees travel one spatial step per time step. Clearly, scaling up the resolution comes at the cost of being able to accurately track the behaviour of individual bees.

### 2.4. Pathogens

Three types of pathogens are currently included in Bee++: *Varroa* mites, *Nosema apis* and *Nosema cerenae*. These are arguably the three most common pathogens affecting honey bee colonies [60]. They are also the three pathogens most likely to be factors in colony collapse [6]. Again, for computational reasons, it is not feasible to track these pathogens individually, but detailed pathogen-specific behaviour is feasible as described in Appendix A.2. The behaviour of these pathogens is described in detail in Appendix A.2.

## 3. Results

**Figure 3:**
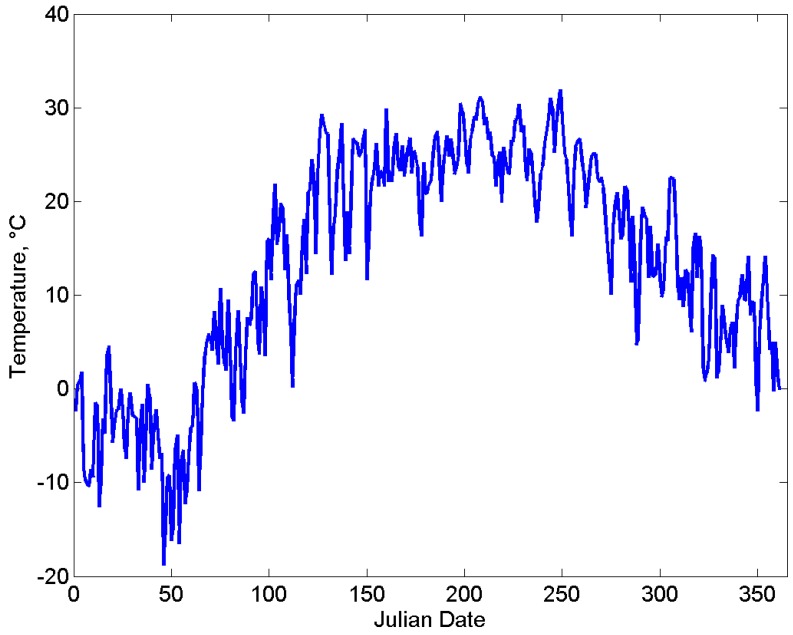
Daily temperature highs for London, Ontario, Canada, for the 2015 calendar year, measured at weather station London CS. Data provided by Environment Canada.

We illustrate the use of Bee++ by simulating a single hive, with several nectar and pollen sources, over the course of one spring/summer season. Temperature data were obtained for 2015 from London CS Station at London International Airport (YXU) in London, Ontario, Canada, as shown in Figure 3. Simulations were run with constant availability of pesticide-free food so as to highlight the dynamics of the bees themselves without potential environmental pressures.

Table 1 shows the key parameter values used in the simulations. A comprehensive list of all parameter choices are given as the default values in Bee++ in the file Parameters.cpp.

**Figure 4:**
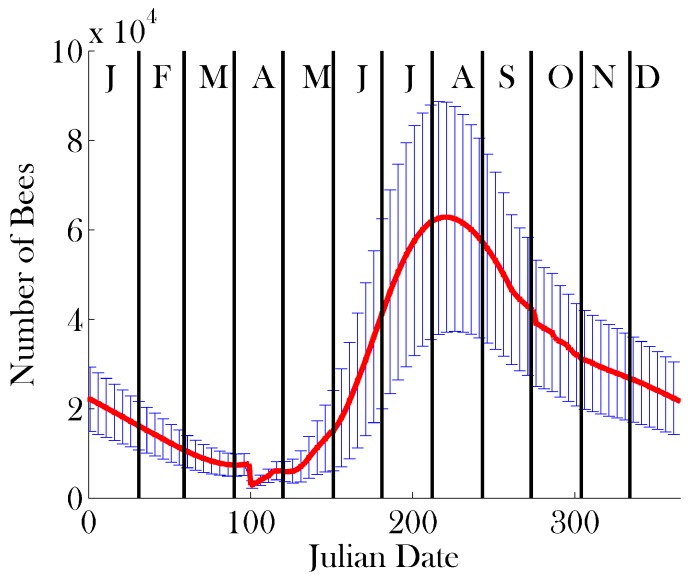
Results of sensitivity analysis on total colony size: mean (red line) ± one standard deviation (error bars). Parameter sets were sampled using Latin hypercube sampling [61] with 10 divisions from the mean values provided in Table 1, ranging ±20%. The parameters on which sensitivity analysis was performed appear in bold in Table 1.

**Figure 5:**
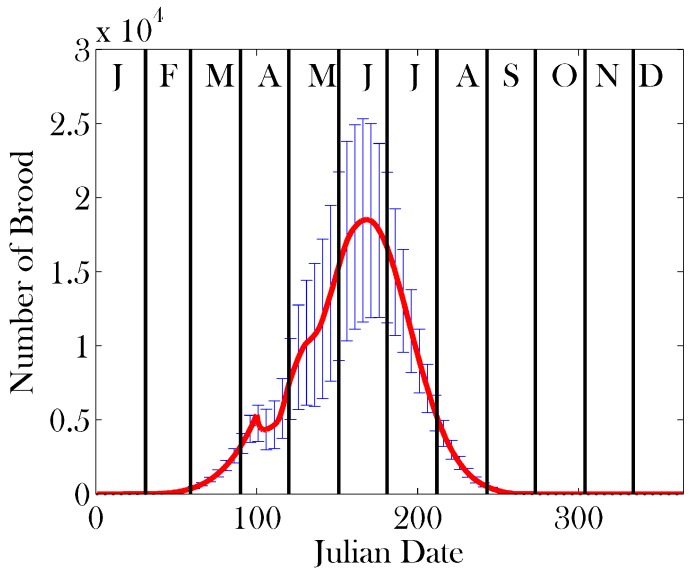
Results of sensitivity analysis on brood size: mean (red line) ± one standard deviation (error bars). Parameter sets were sampled using Latin hypercube sampling [61] with 10 divisions from the mean values provided in Table 1, ranging ±20%. The parameters on which sensitivity analysis was performed appear in bold in Table 1.

**Figure 6:**
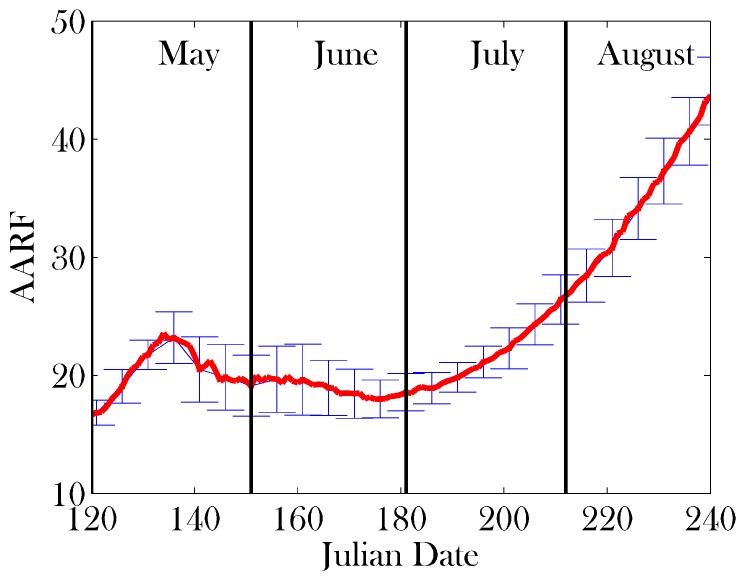
Results of sensitivity analysis on Average Age of Recruitment to Foraging (AARF): mean (red line) ± one standard deviation (error bars). Parameter sets were sampled using Latin hypercube sampling [61] with 10 divisions from the mean values provided in Table 1, ranging ±20%. The parameters on which sensitivity analysis was performed appear in bold in Table 1.

Sensitivity analysis was performed on a subset of 17 parameters, which we believe are crucial to the function of a healthy colony. We varied these parameters by ±20% around the mean values given in Table 1. The parameters used are denoted in bold in Table 1. Given the large parameter space this creates, we use Latin hypercube sampling [61] with 10 divisions in order to obtain an illustrative sample of the parameter space. The sample sets used are represented in Table 2. The results of the sensitivity analysis on the total colony size, brood size, and Average Age of Recruitment to Foraging (AARF) are shown in Figure 4, Figure 5 and Figure 6, respectively.

**Figure 7:**
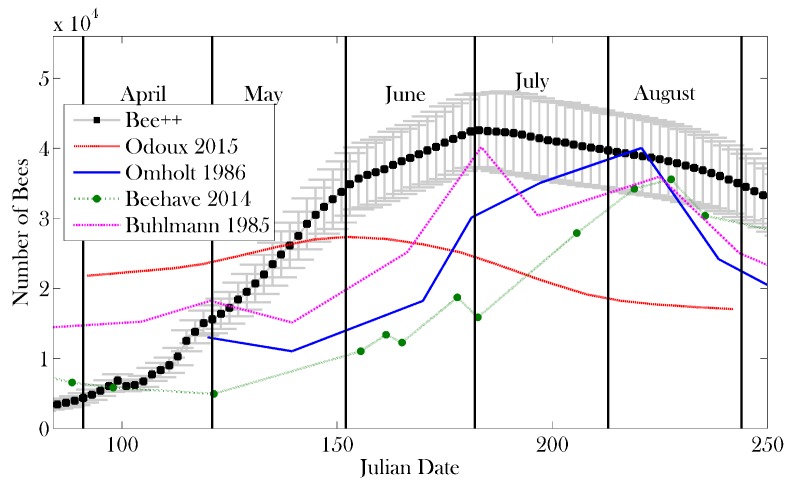
Total bee population from early spring to late summer using data from 10 simulations with the same parameter set (mean in black circles, grey error bars show ± one standard deviation). These 10 simulations begin after the model has simulated 12 years of data, thereby representing the colony size and distribution of a well established colony. Results from the model are compared with those of other studies [52,70,71,72]. The simulation agrees qualitatively with previous models (Omholt) [71], observation (Buhlmann) [72], and simulation (Beehave) [52]. Data from Odoux [70] were obtained by model fitting, using measurements from many colonies. In the Bee++ example we have assumed constant availability of food in the environment and therefore we see a larger peak population.

**Figure 8:**
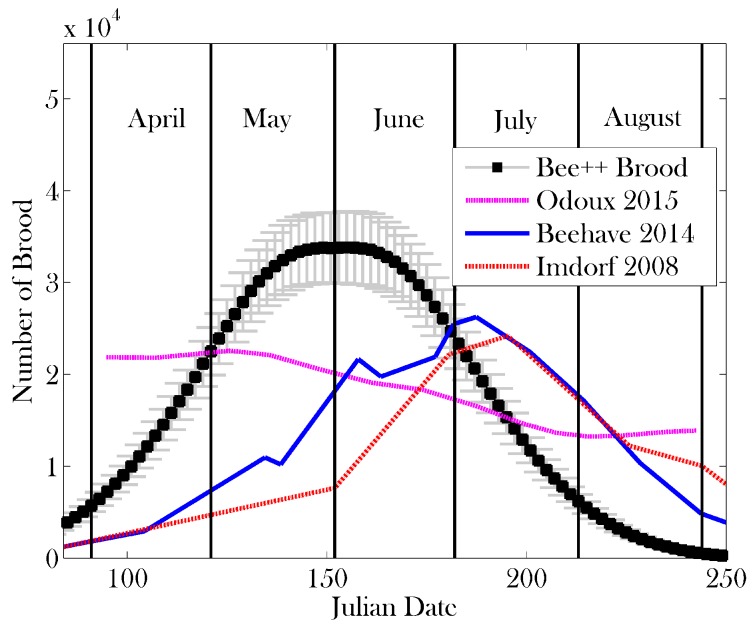
A comparison of brood size obtained using 10 simulations of Bee++ with simulation [52], observed data (Imdorf) [73], and fitted data (Odoux) [70]. Again we have simulated data for a well established colony, and note that the temporal distribution of brood size is influenced largely by the function used to model the laying rate of the queen. As in Figure 7, we see consistent qualitative agreement in all cases except in comparison to [70], which represents fitted data averaged over many colonies. Given the 21 days development time of the brood, the peak in the brood appears roughly 21 days before the peak in adult bee numbers. The simulation in Bee++ assumed constant availability of food throughout the spring and summer. Since brood survival depends in part on the available food, we see a much smoother curve.

Figure 7 and Figure 8 show the results of the simulation for comparison to other data from the literature, namely [27,52,70,71,72]. The data from [70] are fitted using a series of measurements related to colony size; [52] is simulated data; [71] is model data; and [72] is measured data. Given that the climate in London, Ontario, is different from that of, say, northern France in the case of [70] or Hertfordshire in the case of [52], we would like to highlight the qualitative similarities between these results and extant data.

In addition, note that Bee++ is able to capture the decreasing spring population as seen in [25]. The results suggest that this drop is caused by a combination of the increased average age of the population within the colony at the end of winter, and the temperature oscillations around the threshold temperature. The addition of time-dependent food availability would surely further exacerbate this effect.

**Figure 9:**
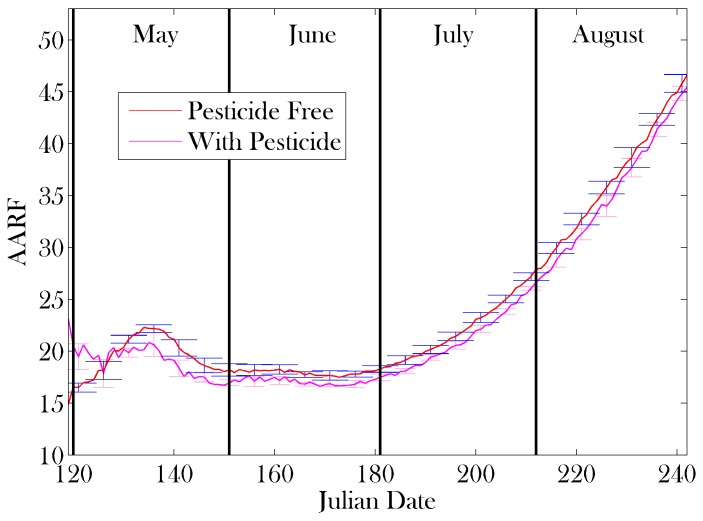
The average age of recruitment to foraging calculated in 7 simulations without pesticides (red line) and a simulation with pesticide treated plants (magenta line) through the summer months. Error bars represent one standard deviation. As mentioned in the text, pesticide exposure in this example affects the navigation abilities of the foragers but does not affect their lifespan, or the age at which they are recruited, thus it may be studied independently from other confounding effects. We see that the AARF is robust against pesticide exposure. We also see that an age of recruitment between 20–30 days corresponds to a healthy colony. An AARF that is above this range could indicate a lack of new brood or problem in the brood, as the increase in the AARF corresponds to the decrease in the brood population in Figure 8. An AARF below this range (as seen in [10,13,74]) can indicate an external stressor (in this case the added stress of early spring on the colony).

**Figure 10:**
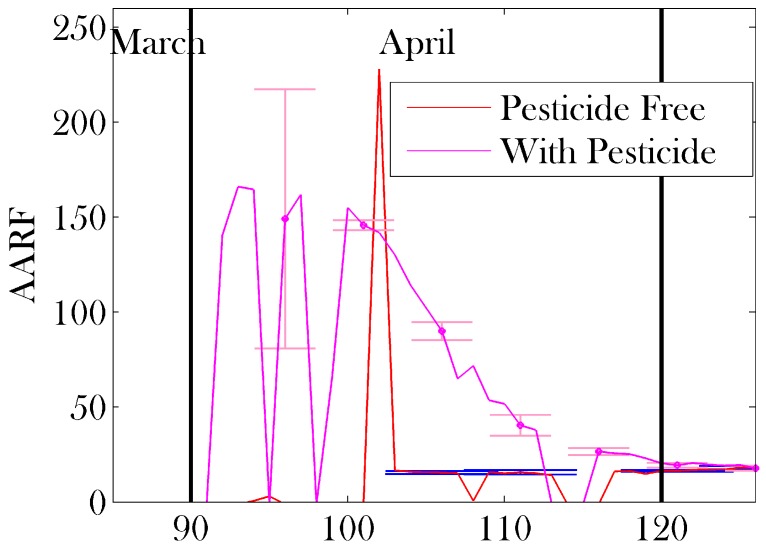
The average age of recruitment to foraging calculated in 7 simulations without pesticides (red line) and a simulation with pesticide treated plants (magenta line) through spring. Error bars, blue and pink respectively, represent one standard deviation. Compared with the results in Figure 9, the value of AARF is here seen to be generally higher and highly volatile in April. This is caused by large swings in the ambient temperature during this time, the ageing of bees and the absence of new bees during winter. Thus AARF is not a useful metric of the health of the colony during early spring.

Figure 9 and Figure 10 show the Average Age of Recruitment to Foraging (AARF). This metric is highly variable when the temperature is close to the threshold in early spring (Figure 10), but stabilizes as the temperature remains above the threshold temperature, and increases steadily as the colony becomes stronger in numbers (Figure 9), eventually leading to a situation in which foraging is an ‘end of life’ activity. The two lines in the plot compare the AARF for a colony that is not exposed to pesticides, with one that is consistently exposed to pesticides through the local plant life. In this example, we remove all effects of pesticides except the pesticide’s ability to disrupt the navigation abilities of the foraging bees. We see that the AARF is robust against this particular effect of pesticides on a colony.

**Figure 11:**
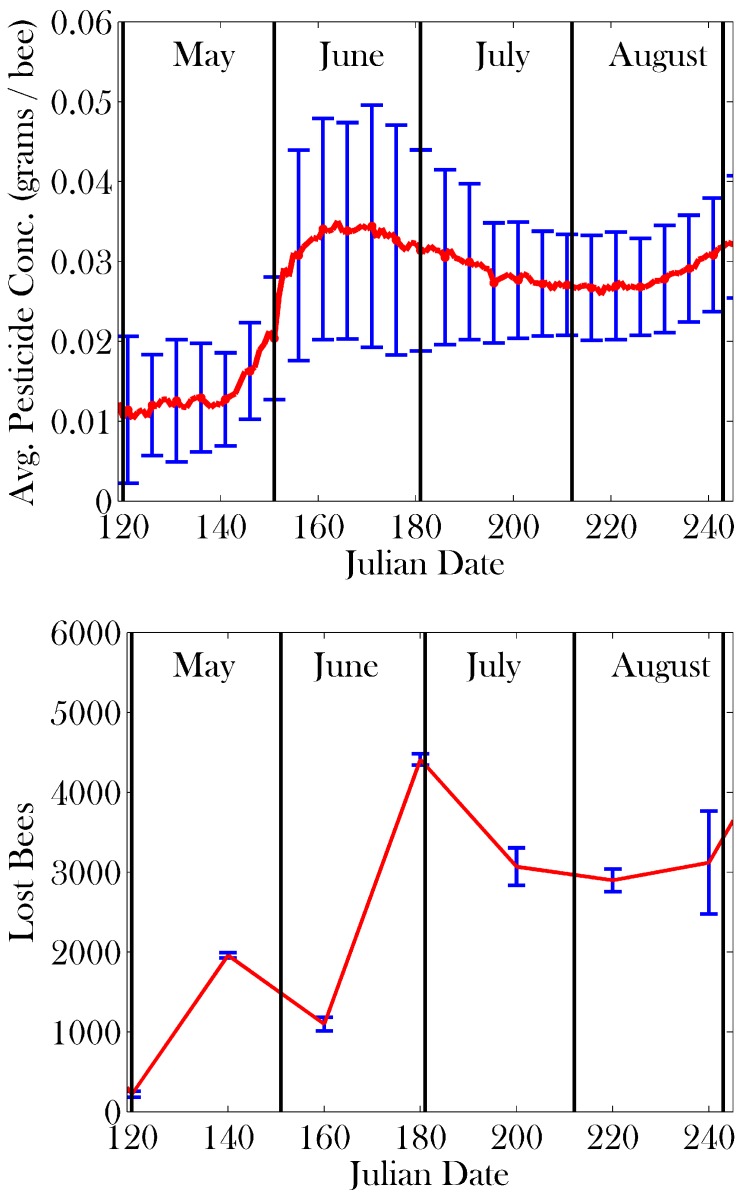
Toxicity of foragers and its effect on navigation over time. Seven simulations were used. Error bars represent one standard deviation. The top plot shows the metabolic efforts of the bees are not enough to completely flush the pesticides from their system. We see that once pesticide concentration in a bee is high enough to force higher metabolic effort, there is a decrease to a more stable concentration. The bottom plot shows the correlation between pesticide exposure and its effects on navigation. Plotted is the current date versus the number of bees lost in the previous 20 days. Bees are considered lost if they die outside a radius of nine patches from the hive (food is at most nine patches away). This plot highlights the functionality of Bee++ as well as how pesticide exposure may explain one possible symptom of CCD.

**Figure 12:**
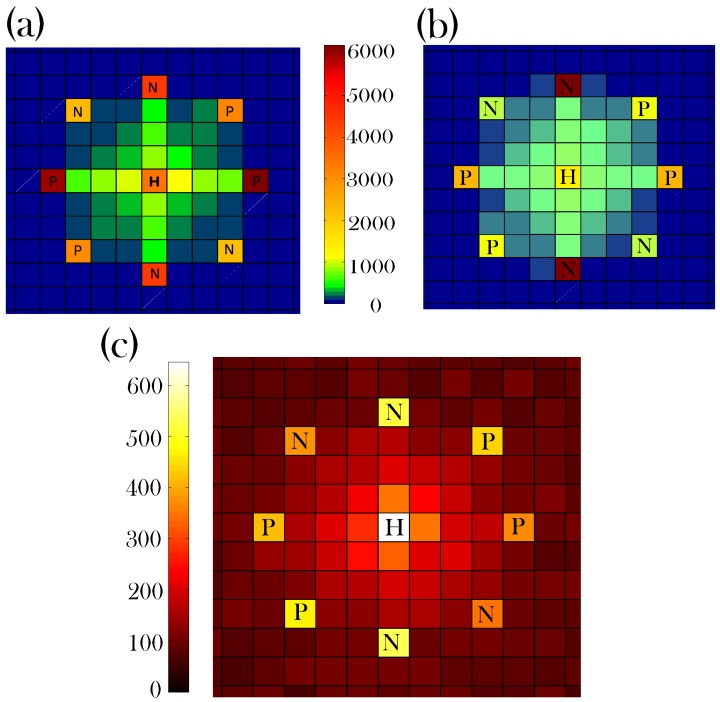
Number of dead foragers found in each patch of a 25×25 grid of 4m × 4m patches totalled between February and September. The total number of dead foragers in this time is 55,283, 55,592 and 56,426 in (**a**–**c**) respectively. The pollen and nectar sources (in patches labelled P and N, respectively) are assumed to be constant throughout the year. The hive itself is labelled H. The colour resolution is roughly 100 bees in Panels ((**a**,**b**) and 10 bees in Panel (**c**). Panel ((**a**) shows a simulation with no pesticide exposure but with normal navigation error ε=0.1. In Panels (**b**,**c**). this navigation error is increased because of pesticide exposure to a maximum of 0.35 and 1.0, respectively. In Panel ((**a**), there are fewer than 10 bees in each blue cell, and in Panel (**b**), there are between 10 and 30 bees in each blue cell. The effect of pesticide on the bees is cumulative. In early spring, there is less build up of toxins within individual bees and thus their navigation is better than it is in late summer.

Tracking toxicity levels in bees individually allows Bee++ to explore and confirm the effects of pesticides on a colony. Figure 11 shows a time course of the average pesticide concentration in the foraging population as well as the number of bees who have become lost in the last 20 days as a result. As a liberal estimate, we assume that bees only die of pesticide exposure if they consume their body weight in pesticide. In Panels (b) and (c) of Figure 12, we introduce pesticides in the local environment which have the effect of interfering with honey bee navigation. In the third panel we note that a far more uniform distribution of honey bee deaths outside the hive results, indicating that navigation even at these short distances has been affected by consistent exposure to pesticides, even though the doses remain sub-lethal. In the case illustrated in Panel (c), parameters were set such that bees with high toxicity can lose any semblance of navigational ability (i.e., max(ε)=1). In this situation, food stores become depleted as many bees cannot find their way back to the hive, and the colony goes extinct in late spring. Figure 12b shows a more realistic case in which max(ε)=0.3. Here, the dead bees are contained within the ‘boundary’ of the food sources, but they are more evenly distributed.

The spatial distribution of bee deaths can be easily tracked in an agent-based simulation; this distribution is of interest since a lack of dead bees around the hive is a symptom of Colony Collapse Disorder [75,76]. Figure 12a shows the spatial distribution of bees that have died, while away from the hive, between February and September of the simulated year. We see that the highest concentration of dead bees is near the food sources as this is where foragers spend the majority of their time outside the hive. Note that bees tend to find their way back to the hive fairly well; although not visible in the colour resolution of the figure, fewer than 10 bees have died in each spatial cell beyond the food sources, over the course of the season.

## 4. Discussion

We present a detailed, agent-based simulation package for honey bee colony dynamics, which is broadly configurable and can be adapted to address a wide range of investigations. In the Results, we demonstrate the behaviour of the model at default parameter values, predicting colony dynamics that are comparable to those predicted by other models and simulations [10,27,52]. The larger peak population size obtained in our simulations is due to the idealized condition that food is plentiful and constant, allowing bees to successfully forage from early spring through late fall; this is configurable in the model. Moreover, the peak population predicted by Bee++ is well within the range of realistic managed colonies [77]. In the simulated data, this peak appears in mid-summer, consistent with observed bee behaviour [78]. Field observations indicate that variation in the timing of this peak is quite large, with some honey bee populations peaking as early as late spring [79], depending on the local climate.

Sensitivity analysis shows that the model behaves as expected under a range of biologically reasonable conditions. Parameters not tested here, such as those associated with toxicity and the transmission rates for different parasites, will clearly also affect the dynamics of the colony. Sensitivity analyses for these can be carried out by individual researchers after their default environmental and colony parameters have been chosen. The framework for sensitivity analysis is built into the source code and can easily be modified to accommodate a detailed sensitivity study for each research question.

The flight patterns of honey bees, and the factors impacting their navigation are complex and quite involved [80]. The navigation used for the bees in Bee++ is macro scale navigation, given the size of the patches intended for use (≥1 m${}^{2}$). This means that the bees are moving toward a general target, and their individual movements between flowers is not modelled here. Models of movement between individual flowers have been well studied [38]. Future work entails optimizing the model so that navigation between individual flowers can be included. Moreover, displaced or lost honey bees have been shown to use a number of mechanisms to search for the hive, which can be modelled as a Lévy flight [39]. These mechanisms could also be considered in future extensions of the flight mechanisms in Bee++.

Figure 8 shows that the brood population of a healthy colony peaks in early June, consistent again with experimental findings [81]. Qualitatively, we see the same brood-rearing dynamics as predicted by other models. The measurements provided by [70] differ qualitatively from the other data presented in Figure 8. One potential explanation for this discrepancy is that the results of [70] are calculated using 208 different colonies, over four years. Different weather patterns over the 4 years and across the different locales may force the colonies to peak at different times of the year, potentially obfuscating the peak experienced by any one colony.

Figure 9 predicts that, when the pesticide-free colony population is at its strongest, the average age of recruitment to foraging is between approximately 20 and 30 days old. This is in full agreement with previous results [10,18,53,74]. Furthermore, in early spring, when the colony is under increased stress, we see a lower AARF, consistent with the predictions of [10], which correlate the AARF and colony health. Our simulations also show that as the number of eggs laid by the queen diminishes in late summer, the AARF increases substantially. This behaviour seems to benefit the colony, as the younger, stronger bees are left in the hive for the incoming winter.

The results shown in Figure 9 indicate that while the AARF can be used to determine certain aspects of colony health (i.e., a high AARF in the fall corresponds to dwindling numbers of the brood, or as an indication of infection [13]), the AARF is *not* a strong metric for the effects of pesticides on the navigational abilities of the worker bees. Results such as those in [74] (accelerated maturation of bees) can also be added to Bee++, further compounding the effects of pesticides on the age of foragers, with likely detrimental results. While the AARF has been previously proposed as a measure of colony health [10,13,18], the source of any change to the AARF is hard to extract from this metric, as many stresses will change its value. Therefore, it is important to know what will and will not effect this metric as some stresses (such as degrading navigational ability) can be detrimental to the colony, but asymptomatic without invasive tests on the bees (such as determining toxicity within the bees). In future work, we would like to study the compounding effects of pesticides, and also investigate the use of time-dependent AARF and other metrics to potentially identify which source of stress is causing the most harm to the bees. One preliminary hypothesis is that high AARF may indicate a problem in the brood, as AARF indeed increases in our model as the brood population decreases.

One of the key benefits of Bee++ is the ability to explicitly track toxins within individual bees, a feature that has not been included in previous studies. With this mechanism, we are able to explore explicit effects of sub-lethal doses of pesticides on honey bees without approximating these effects as an increased death rate. In particular, our model explicitly allows for pesticide exposure to interfere with foragers’ navigational abilities, as has been demonstrated experimentally [8]. When comparing the panels of Figure 12, we see that as pesticides affect navigation more strongly, more bees die at distances further from the hive. Importantly, a lack of dead bees around the hive is one of the symptoms of Colony Collapse Disorder (CCD) [75,76]; our model predicts that an encumbrance on foragers’ navigation ability can help explain this phenomenon. In other words, a loss of navigation ability is one potential explanation for the lack of bee corpses reported in CCD. This inability to navigate in turn causes a reduction in the amount of food in the hive, and thus an increased death rate as the bees are generally weaker.

Given the recent proposal by the U.S. Fish and Wildlife service to declare the rusty-patched bumble bee as an endangered species [82], an accurate, parameter-rich model of bumble bee dynamics may be crucial in developing policies to protect this species. The source code of Bee++ can be modified to perform such a task, as bumble bee populations share a very similar social structure to honey bees. We hope to do so in the near future.

As well, our future work includes plans to modify the model to run on parallel processing architectures. With the exception of the waggle dances, each bee makes a decision at each time step independent of other bees, thus it should be possible to create a more efficient code by allowing (ideally) all bees to make decisions simultaneously.

Overall, Bee++ has the ability to create realistic simulations of honey bee colonies by incorporating a wide range of parameters and potential interactions. The object-oriented nature of the model implementation in C++ allows for easy modification of the source code so that the model can easily be improved and expanded as more experimental data become available. The simulations of Bee++ can be used to study the potential underlying causes of honey bee colony decline such as the effects of biodiversity, disease, predation and toxicity on colony dynamics. The effects of preventive measures (such as optimal plant diversity, crop density or anti-viral treatments) or recovery plans (such as manual feeding or colony transplant) can thus be studied before costly implementation.

## Figures and Tables

**Table 1:** Parameter values and source references.

**L**	maximum rate of egg laying	1500 eggs/day	[62]
w	number of hive bees for 50% egg survival	1000 bees	
b	mass of food stored for 50% egg survival	500 g/day	[18]
$a_{m}$	age at which hive bees begin brood care	4 days	[48]
$a_{T}$	age at which hive bees end brood care	16 days	[48]
$a_{R}$	minimum age of recruitment to foraging	4 days	[63]
k	age at which rate of recruitment is 50% of max.	10 days	
*α*	maximum rate of recruitment	1 /day	
$\frac{1}{\sigma }$	maximum fraction of colony that can be foraging	$\frac{1}{3}$	[10]
$d_{n}$	natural death rate of foragers outside the hive	equation (10)	[64]
$c_{n}$	nectar gathered per day per forager	0.03 g/day/bee	[65]
$c_{p}$	pollen gathered per day per forager	0.016 g/day/bee	[65]
$\gamma_{H}$	daily food requirement per hive bee	0.08 g/bee	[66,67]
$\gamma_{F}$	daily food requirement per forager	0.13 g/bee	[66,67]
$\gamma_{D}$	daily food requirement per drone	0.1 g/bee	[66,67]
$\gamma_{Bp}$	daily pollen requirement per uncapped brood cell	0.067 g/bee	[66,67]
$\gamma_{Bn}$	daily nectar requirement per uncapped brood cell	0.018 g/bee	[66,67]
K	Shape parameter for exit probability of forager	30 min	
$t_{R}$	Minimum time in hive	1 h	
$t_{E}$	Time forager spends searching for target	15 min	
$t_{H}$	Maximum flight time	45 min	
$T_{W}$	Temperature at which foraging begins	10 ${}^{\circ }$C	[34,35]
$T_{I}$	Ideal ambient temperature *	25 ${}^{\circ }$C	[34,35,54]
$T_{1}$	Minimum foraging temperature	10 ${}^{\circ }$C	[34,35,54]
$T_{2}$	Maximum foraging temperature	40 ${}^{\circ }$C	[36]
$x_{D}$	Rate at which pesticides are metabolised	3.33 /day	[50,51]
${\hat{C}}_{P}$	Concentration of pesticide in nectar	1.9 ng/g	[68,69]
$\varepsilon_{base}$	Error in forager navigation	0.1	
*φ*	Scaling of pesticide effect on navigation	10	

* Bee++ assumes there is always some metabolic heat being generated by the bees; therefore, we set the ideal temperature lower than the measured ideal hive temperature in [54]. Bold indicates parameters for which sensitivity analysis was performed..

**Table 2:** Parameters used in 10 simulations for sensitivity analysis. Each column represents one simulation. Mean values provided in Table 1.

	% Change from Mean									
*k*	1.152	−5.9516	13.53	11.145	−10.583	−1.5224	−12.415	6.49	−17.051	19.035
*w*	−14.492	−18.092	2.3472	15.129	16.566	−11.444	6.8644	−2.4616	−7.118	8.1808
*b*	12.322	−17.539	7.3496	−15.187	−4.4552	−8.6352	10.577	16.609	−2.3312	1.6552
*K*	16.297	13.7	7.96	−11.238	9.5648	−17.92	−5.0736	−13.032	−1.924	3.0908
$a_{R}$	19.806	−12.677	−5.0336	7.2084	1.5772	−11.295	8.7576	12.791	−18.832	−3.4376
$a_{m}$	−19.124	3.1848	−3.9732	15.626	−14.602	7.1392	−4.9756	18.641	11.208	−10.027
$t_{H}$	−3.8032	−9.5784	17.662	−6.426	-14.858	2.394	12.565	8.318	4.9968	−17.142
$\gamma_{F}$	−7.1872	15.429	−10.018	1.5572	−14.815	−17.533	−2.9148	4.4508	19.777	11.447
$\gamma_{H}$	−19.452	−1.6868	−9.6452	−7.8364	16.999	0.076	7.066	11.615	−13.538	13.999
$\gamma_{D}$	−18.281	12.094	10.028	6.3964	−15.98	−9.044	−6.6612	−3.8572	17.315	2.8032
$\gamma_{Bp}$	13.876	−0.006	16.465	−17.618	2.7952	−11.802	−5.8424	10.85	−12.338	5.6712
$\gamma_{Bn}$	7.3876	15.708	−10.322	−13.148	18.552	−2.8992	−7.4332	2.6084	−19.847	8.1856
$t_{E}$	−4.824	12.927	9.538	0.3244	−14.41	-2.8084	17.025	−9.5404	−17.006	7.8528
$t_{H}$	−5.8884	−10.58	6.884	13.928	11.017	−17.19	−14.602	−3.5656	16.556	3.1604
$c_{N}$	10.404	12.448	−5.026	−19.867	1.5232	7.3388	−11.305	−2.6228	−14.186	18.995
$c_{P}$	−0.1608	3.0664	−5.4444	−14.534	−19.944	15.171	8.9716	−11.545	18.111	7.3644
*L*	8.7564	18.094	−16.465	−7.5028	−2.5408	7.6108	−11.634	15.86	−12.159	0.0456

## Appendix A

### Appendix A.1. Drone Dynamics

Drones are born of the unfertilized eggs laid by the queen [56]. Their main duty in life is procreation with a queen of another hive. Upon success, the drone dies [56]. The drones make two decisions in their lifetime. With probability

(A1) $$p_{L}=\max\left(e^{t-t^{*}},\;1\;\right)$$

the drones will leave the hive in search of a mate. Here, $t^{*}$ represents the optimal time of year for mating. The drones then move in much the same fashion as foragers, with their target being the mating area. Those that become lost will return to the hive, and those that reach the mating area will successfully mate with a queen with probability

(A2) $$\begin{array}{c} p_{M}=\min\left(\frac{N_{Q}}{N_{D}},\;1\right) \end{array}$$

where $N_{Q}$ is the number of queens present in the mating area and $N_{D}$ is the number of drones present. The drones do not mate with their own queen (as they are genetic clones of the queen). The mating process is built into Bee++ so that realistic numbers of drones can be modelled (since mating is followed by death of the drone). As well, having this functionality is the first step to creating a model and simulation package with interacting colonies. At present, multiple colonies are not implemented in the model and therefore the position of any mating sites and the number of queens present at any given time must be set manually by the user. Nonetheless the drones are an important part of a honey bee colony, as they can be a source of pathogens as well as a drain on the food stores and can account for up to 15% of the total population [83].

### Appendix A.2. Pathogen Dynamics

Both sub-types of the microsporidian *Nosema* reproduce within the epithelial cells of live bees, which in turn causes dysentery, forcing bees to expel waste within hive cells where spores will be picked up by bees, which clean contaminated cells [84]. *Nosema ceranae* is reported to affect foragers disproportionately, killing them away from the hive at an unsustainable rate [17]. There is also evidence that spores can be transmitted to bees through food stores [60]. With this in mind, we posit that bees that clean cells (i.e., nurses) are most likely to be infected, while secondary mechanisms may exist through direct bee to bee transmission and food stores. Therefore, we constrain the rates of transmission of either type of *Nosema* spore to foragers, drones, maintenance bees and nurses ($\beta_{F}$, $\beta_{D}$, $\beta_{M}$ and $\beta_{N}$ respectively) through the condition

(A3) $$\begin{array}{c} \beta_{F}\approx \beta_{D}\approx \beta_{M}<\beta_{N}. \end{array}$$

Since hive cells are often soiled during the winter, when honey bees cannot leave the hive to defecate, the transmission of *Nosema* spores is temperature dependent. Presently Bee++ does not consider a spatially explicit interior to the hive, therefore we cannot track individual brood/food cells and instead treat the pathogen populations and bee populations as well mixed.

Each infection rate will then depend on the number of spores in the environment (which in turn depends on the number of infected bees), as well as susceptibility of a particular bee to infection (pesticide exposure may increase susceptibility [19]). In other words, we define the time-dependent infection rates, $p_{I}$, as

(A4) $$\begin{array}{c} p_{I,i,j}=\beta_{i}\left(P,\vec{I},i\right)\frac{S_{j}}{s+S_{j}} \end{array}$$

where $\beta_{i}$ is a function dependent on the toxicity level *P* of the bee, the number of infections *I* it has acquired that may be compromising its immune system, and the caste of the bee, *i*. $S_{j}$ is the number of free *Nosema* spores of type *j* (*apis* or *cerenae*) and *s* is a shape parameter. This infection through free spores is the main transmission method for both types of Nosema, and the one that our model currently considers. Of course, future work can expand the model to include secondary transmission routes.

The population of free spores of type *j*, $S_{j}$, is governed by the differential equation,

(A5) $$\begin{array}{c} \frac{dS_{j}}{dt}=\sum_{i}B_{S,i,j}I_{i}-CN_{N}. \end{array}$$

where $B_{S,i,j}$ is the shedding rate of Nosema spores of type *j* by one infected bee of type *i* and $I_{i}$ is the number of infected bees of type *i*, *C* is the rate at which nurses can clean the cells, and $N_{N}$ is once again the total number of nurses.

There is evidence that the fungicide, which prevents *Nosema apis* infection within honey bee colonies, may actually allow *Nosema ceranae* to thrive [85]. Therefore we thought it imperative to include both possible infections in our model, as well as the *N. apis* treatment, fumagillin, as a toxin. In this way, our model allows one to explore the trade-offs between these three very important, and evidently lethal stressors.

*Varroa* mites on the other hand, reproduce in the sealed brood cells [86]. Within the cell, the mite will lay female and male eggs (usually one male and five female [12]. The mites mate within the sealed brood cell [87], and the female mites emerge with the larvae, and attach themselves to nearby adult worker bees [86]. The mites feed on the blood of the adult worker bees, and use them as a means to disperse to new brood cells where they may reproduce [88].

*Varroa* has been observed to act as a vector for viruses such as Deformed Wing Virus (DWV) and Acute Paralysis Virus (APV) [87,89]. Therefore, our *Varroa* mites are modelled as three types, $V_{i}$, depending on whether they do not carry any virus, are carriers of DWV, or carriers of APV.

*Varroa* mites feed on adult bees for approximately 4 to 5 days [88] before entering a brood cell that is about to be capped. Therefore, we keep track of the number of days a bee has had a mite attached. When a mite has reached reproductive maturity, with probability $p_{E}\left(t\right)$ the mite will enter a viable brood cell. Once the mite enters a brood cell, the adult bee is no longer considered infested with a mite. The number of mites that leave their adult bee hosts are exactly the number of brood cells that end up inhabited by a *Varroa* mite. We recognize that this assumes that more than one mite cannot occupy the same brood cell. We use this assumption first on the basis of empirical data [88] and, second, in order to make the problem tractable without explicitly tracking the spatial movement of the bees within the hive. We further assume that all mites either enter a brood cell or die after 5 days of attaching to a host.

The number of new *Varroa* mites created by one infected brood cell as the new bee emerges is

(A6) $$\begin{array}{c} V_{new}=r_{V}B_{I} \end{array}$$

where $r_{V}$ is the average number of female mites produced by one foundress (i.e., an egg-laying mite), and $B_{I}$ is the number of infected brood cells. We further assume that upon hatching, the new female mites find a host bee or die. Therefore the number of infected bees is directly proportional to the number of surviving emerging mites.
